# Defining and targeting mechanisms of eosinophilic inflammation in a new era of severe asthma treatment

**DOI:** 10.1172/JCI182410

**Published:** 2024-06-17

**Authors:** Joshua A. Boyce, Stephanie C. Eisenbarth

**Affiliations:** 1 Department of Immunology, Harvard Medical School, Boston, Massachusetts, USA.; 2 Division of Allergy and Immunology, Department of Medicine, and; 3 Center for Human Immunobiology, Northwestern University Feinberg School of Medicine, Chicago, Illinois, USA.

## Abstract

Studies using mouse models of airway disease have advanced our understanding of the mechanisms driving eosinophilic airway inflammation and demonstrated potential therapeutic targets in asthma.

Eosinophilic mucosal inflammation of the airways is a long-recognized feature of most cases of asthma. Elevated blood eosinophil counts and serum IgE levels continue to serve as markers of type 2–high (also known as T2 or atopic) asthma, named for the involvement of type 2 cytokines. Although type 2 inflammation was initially thought to drive all forms of asthma, more recent work has identified other pathophysiologic mechanisms with differing clinical phenotypes, and identification of these endotypes now helps tailor therapeutic approaches. The recognition that eosinophil counts in bronchial biopsies from people with asthma correlate with disease severity (1) spurred interest in mechanisms that control eosinophil development and tissue recruitment. The development of mouse models of allergic airway diseases in the early 1990s was instrumental in dissecting out the relative roles of each of the molecular signals and identifying the relevant mechanistic links between cytokines and cellular pathophysiology (Figure 1). The identification of cytokines facilitated the discovery of polarized Th1 and Th2 lymphocyte populations in mice that serve divergent functions in adaptive immunity (2). Subsequently, putative Th2 lymphocytes expressing mRNA for IL-3, IL-4, IL-5, and GM-CSF were discovered in the bronchoalveolar lavage fluid of patients with mild, steroid-naive atopic asthma (3). These early studies pointed to the role of polarized T cells, and the cytokines and chemokines they produce, in driving features of asthma, including eosinophilia and elevated serum IgE.

## Cytokine regulation of eosinophil development and recruitment

The three cytokines IL-3, GM-CSF, and IL-5 that are encoded within a cluster of genes on human chromosome 5q31, referred to as the type 2 cytokine gene cluster, demonstrated eosinophil growth factor activity from human hematopoietic progenitor cells in colony-forming assays (4). Each of these cytokines binds heterodimeric receptors composed of ligand-specific α subunits and a common β subunit, the latter responsible for signal transduction. Although GM-CSF and IL-3 act on multiple lineages, IL-5 acts selectively on eosinophils due the relatively restricted expression of the α subunit of the IL-5 receptor (IL-5Rα). All three cytokines primed human blood eosinophils for activation and sustained their survival in vitro and rendered them resistant to apoptosis induced by glucocorticoids (5). IL-5 promotes selective eosinophil egress from the bone marrow and induction of lung tissue eosinophilia in allergen-sensitized and -challenged mice. The relative specificity of this receptor for eosinophils among circulating granulocytes suggested monoclonal antibodies against IL-5 and IL-5Rα as a strategy to mitigate eosinophil-driven pathology in asthma. Ultimately, this goal was realized (6).

As with all circulating leukocytes, eosinophil recruitment to the tissues requires rolling on the endothelium followed by firm integrin-mediated adhesion and subsequent transendothelial migration. Two additional type 2 cytokines encoded by genes on human chromosome 5q31, IL-4 and IL-13, signal through heterodimeric receptors that incorporate the IL-4 receptor α subunit (IL-4Rα). Together, these cytokines are responsible for polarization of naive T cells to Th2 cells, IgE class switching by B cells, and activation of stromal cells. This latter function includes upregulation of endothelial VCAM-1, the ligand for the α_4_β_1_ integrin heterodimer (also referred to as very late antigen 4, VLA-4) (7), and expression of a family of chemokines, several of which signal through eosinophil-associated CCR3. Eosinophils express VLA-4 and CCR3, whereas neutrophils do not, supporting the hypothesis that these IL-4Rα–driven pathways contribute to selective eosinophil tissue recruitment by regulating adhesion and chemotaxis pathways that act in concert with IL-5. These hypotheses were substantiated by genetic deletion models of allergic airway inflammation in mice that appeared in the 1990s.

## Antigen-induced models of asthma in mice

Mouse models of eosinophilic pulmonary inflammation induced by systemic sensitization to innocuous proteins (most typically chicken egg OVA) induce a strong Th2 response and airway hyperresponsiveness (AHR). These models provided a platform to directly test the roles of type 2 cytokines in asthma pathogenesis. In a seminal study published in the *JCI*, Gonzalo and colleagues used several knockout strains of mice and blocking antibodies to understand the requirements for eosinophilic airway pathology (8). They concluded that mature CD4^+^ T cells, but not CD8^+^ T cells, were necessary to drive eosinophil recruitment to the airway and demonstrated essential roles for ICAM-1 and VCAM-1. A blocking antibody against eotaxin-1/CCL11 inhibited eosinophil recruitment by approximately 50%, reflecting the redundancy of the chemokine system and/or additional chemoattractants such as lipid mediators. Surprisingly, T cell–deficient mice showed wild-type expression of CCL11 and other chemokines, in retrospect perhaps due to IL-4 or IL-13 provided by non-CD4^+^ T cells, such as innate lymphoid cells or mast cells. These studies revealed that the IL-4/IL-13 and IL-5 pathways, provided at least partly by CD4^+^ Th cells, act in concert to orchestrate eosinophilic airway inflammation.

Although mouse models are valuable, differences in genetic background and protocol could yield discrepant results, especially as they relate to the regulation of AHR and the role of eosinophils in this process. Studies appearing in 1996 reported that AHR to methacholine and lung eosinophilia in C57BL/6 mice were markedly attenuated by targeted deletion of the IL-5 gene (9); in contrast, monoclonal antibodies blocking IL-4, but not those blocking IL-5, attenuated AHR in OVA-sensitized and -challenged BALB/c mice (10), even though anti–IL-5 effectively blocked airway eosinophilia. The impact of IL-4 blockade was only evident when it was administered during the sensitization phase of the model, in retrospect perhaps due to a requirement for IL-4 to induce polarization of naive T cells to a Th2 phenotype. Mast cells and IgE, which are consistently present in airway tissue, were necessary or dispensable for AHR, depending on the protocol used for sensitization (11). Two independent studies demonstrated that IL-13 was necessary and sufficient to elicit airway eosinophilia, goblet cell metaplasia, collagen deposition, and AHR to methacholine in mice (12, 13). Together, despite differences across models, mouse models supported a focus on therapeutic targeting of the IL-4/IL-4Rα/IL-5/IL-13 pathways for asthma treatment.

## The epithelium and innate lymphoid cells enter the picture

Early classical mouse asthma models induced by systemic sensitization followed by inhalation challenges emphasized the role of adaptive immunity driving eosinophilic pathology. Subsequent studies over the last 15 years revealed parallel systems involving the innate immune system. Barrier cell–derived cytokines called “alarmins,” including IL-33, IL-25, and thymic stromal lymphopoietin (TSLP), are now recognized as major drivers of innate type 2 inflammation in mice and humans. These cytokines convey signals from perturbed epithelial barrier cells to innate effector cells, including a tissue-resident lymphocyte population possessing the transcriptional characteristics of Th2 cells but lacking classical antigen receptors, now known as innate lymphoid type 2 cells (ILC2s) (14). When activated by the alarmins or lipid mediators in vivo in mice, ILC2s can drive tissue eosinophilia independently of functional T cells, reflecting their potent productions of IL-5, IL-9, GM-CSF, IL-13, and, in some contexts, IL-4. IL-33 potently activates tissue mast cells, which can make IL-5 and IL-13 (15). IL-33 also activates eosinophils and basophils, the latter of which are a major source of IL-4. In many models of allergic airway disease, the components of the innate type 2 immune system collaborate with the adaptive immune system to amplify and modify pathophysiologic features. Human studies support that these innate immune pathways are relevant to disease, especially in severe asthma.

## Emergence of type 2–targeted therapeutics and beyond

The knowledge gained from studies using mouse models of airway disease advanced our understanding of the mechanisms driving eosinophilic airway inflammation and demonstrated potential therapeutic targets. Two antibodies against IL-5 and one against IL-5Rα are now approved treatments for severe asthma associated with persistent eosinophilia, in addition to glucocorticoid therapy, reducing annualized exacerbation rates and moderately improving lung function (6). The anti–IL-4Rα antibody dupilumab is highly effective for treatment of severe asthma associated with eosinophilia and elevated serum IgE. Monoclonal anti-IgE, which prevents the binding of IgE to the high-affinity receptor for IgE, reduces annualized exacerbation rates in people with severe atopic asthma. The fact that the immune pathways driven by IL-5, IL-4Rα, and IgE only partly overlap highlights the complex and heterogeneous mechanisms that drive disease pathology, even in people with a T2 asthma endotype who have elevated IgE and blood eosinophil counts and increased fraction of exhaled nitric oxide.

Mouse models cannot recapitulate the genetic heterogeneity, physiology, and environmental complexity of human asthma. The evolution of increasingly sophisticated tools and technology (mass cytometry, RNA sequencing) to study human cells and tissues has substantially advanced our understanding of asthma immunopathology beyond the classic “Th2” paradigm. Severe asthma involves especially heterogeneous immunopathology and in many instances involves type 1, type 3, and mixed immune pathways. Recently approved or therapeutic trials of monoclonal antibodies against TSLP, IL-33 and its receptor ST2, or mast cells (6) validate the roles of these upstream drivers of type 2 inflammation in asthma; however, they also display efficacy in people with severe asthma who lack T2 features. Therefore, targeting early steps in the immunopathogenesis of asthma (i.e., those that depend on injured or reprogrammed barrier cells) may provide broader coverage of the downstream immune pathways responsible for driving asthma in cases with and without T2 biomarker signatures. Iterative investigations in patients and mouse models have advanced the asthma field tremendously over the past 30 years; the result is an armamentarium of new therapeutics that alter disease pathophysiology rather than just symptoms and result in a more tailored use of these therapeutics to particular asthma endotypes. Limitations notwithstanding, mouse models have proven invaluable to identifying therapeutic targets that have improved the lives of patients with asthma, a triumph of bench-to-bedside discovery. The applications of newer tools, such as mice with cell type–specific gene deletions, “humanized” mice, and CRISPR/Cas9 gene deletion strategies, to models of airway disease are likely to uncover additional opportunities for therapeutic development.

## Figures and Tables

**Figure 1:**
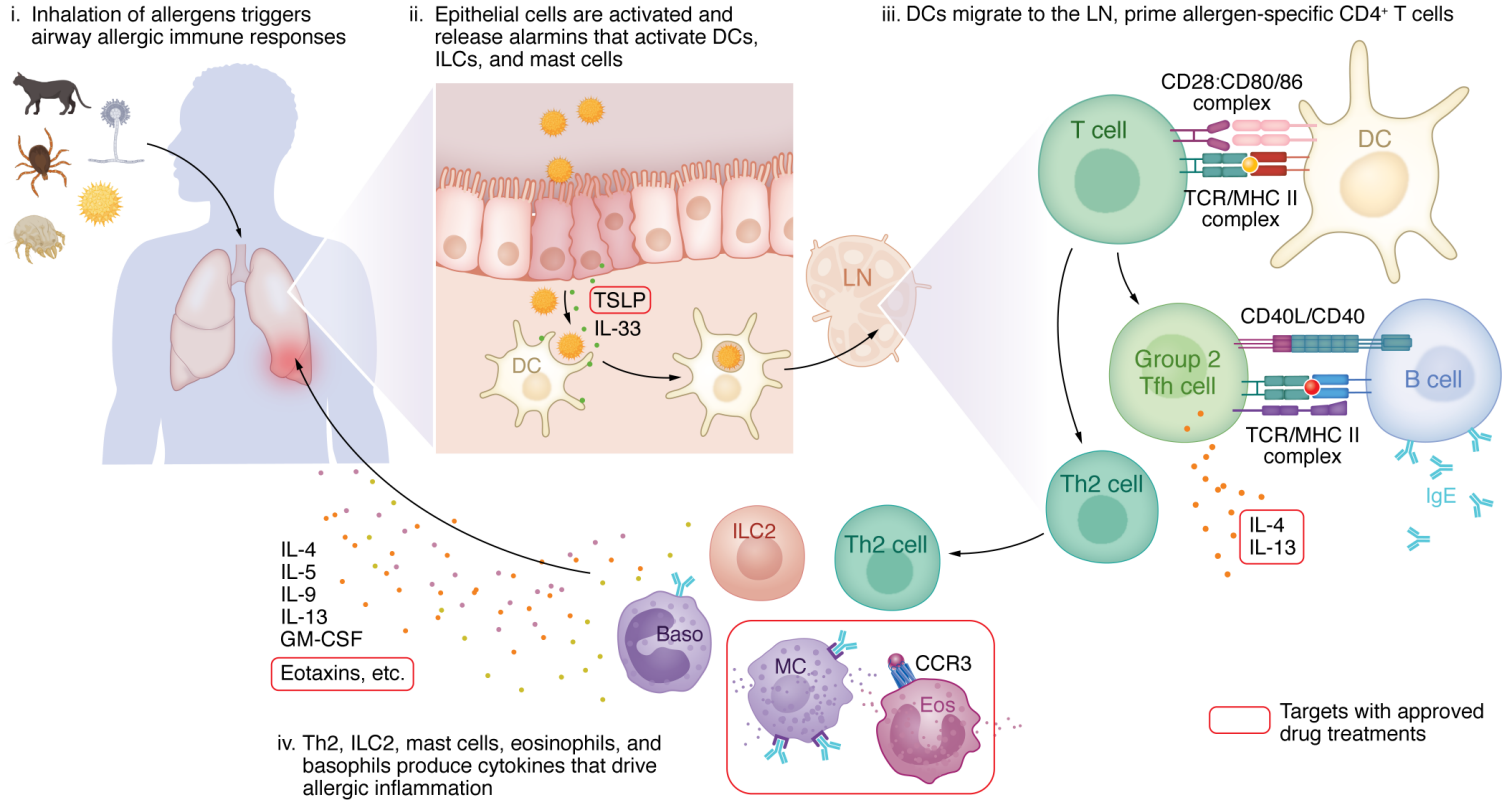
The pathophysiology of atopic asthma. Four stages of T2 asthma inflammation induction are shown. In the first stage, airborne allergens induce inflammation in lung airways through their unique properties. The best-characterized effect of allergens occurs in phase 2, in which the damaged airway epithelium releases alarmins, such as thymic stromal lymphopoietin (TSLP) and IL-33. These factors act on sentinel antigen-presenting cells, in particular local dendritic cells (DCs). During phase 3, DCs carry allergens to lung draining lymph nodes (LNs) and prime naive allergen-specific CD4^^+^^ T cells. Early IL-4 and other signals, many remaining undefined, promote effector Th2 differentiation and group 2 follicular Th (Tfh2 and Tfh13) cells. Tfh cells remain in the LN and promote B cell activation and the production of allergen-specific IgE and other isotypes. In phase 4, effector Th2 cells home back to the lung and, along with innate lymphoid type 2 cells (ILC2s) and lung epithelial cells, produce type 2 cytokines and CCR3 ligands, including CCL11/eotaxin-1, CCL24/eotaxin-2, CCL26/eotaxin-3, monocyte chemoattractant protein-4, and CCL5/RANTES. These cytokines and chemokines promote endothelial and epithelial activation, goblet cell hyperplasia and mucus production, mast cell (MC) and basophil (Baso) activation, and production and recruitment of eosinophils (Eos) to the lung. Approved drugs now target several of these pathways.

## Version 1 06/17/2024

Electronic publication
